# Honokiol Alleviates High-Fat Diet-Induced Obesity of Mice by Inhibiting Adipogenesis and Promoting White Adipose Tissue Browning

**DOI:** 10.3390/ani11061493

**Published:** 2021-05-21

**Authors:** Yanan Ding, Longlin Zhang, Xiaofeng Yao, Haihan Zhang, Xi He, Zhiyong Fan, Zehe Song

**Affiliations:** 1 College of Animal Science and Technology, Hunan Agricultural University, Changsha 410128, China; dingyanan13@163.com (Y.D.); zllwithann@gmail.com (L.Z.); yaoxiaofeng0525@163.com (X.Y.); zhhous@163.com (H.Z.); hexi111@126.com (X.H.); 2 Hunan Co-Innovation Center of Animal Production Safety, Hunan Agricultural University, Changsha 410128, China

**Keywords:** honokiol, high-fat, white adipose tissue, browning

## Abstract

**Simple Summary:**

The browning of white adipose tissue can change the body’s energy distribution and improve the disorder of energy metabolism through the expression of the iconic protein UCP1 to achieve beneficial effects on the body. As a natural plant functional component, polyphenols have been found to induce the browning of white adipose tissue, such as resveratrol and catechin. Honokiol is one of the main components in *Magnolia officinalis*, a traditional Chinese medicine in China. Modern pharmacological tests have proven that, like many plant functional components, honokiol has broad-spectrum antibacterial, anti-tumor, anti-inflammatory, and anti-oxidation effects. However, the effect of honokiol on inducing the browning of white adipose tissue and improving energy metabolism disorder has not been reported. In this study, a high-fat diet-induced obese model was established, and proteomic analysis was performed. It was found that honokiol could promote the browning of white adipose tissue in high-fat fed mice. The AMPK-ACC-CPT1A and C/EBPα-SOAT1 pathways may be the important molecular mechanisms of the browning induced by honokiol.

**Abstract:**

Honokiol (HON) is one of the main biological active components of the traditional Chinese medicine *Magnolia officinalis* and has many health benefits. The aim of this study was to investigate whether HON could alleviate obesity in mice by inhibiting adipogenesis and promoting the browning of white adipose tissue (WAT). C57BL/6 mice were divided into five groups and fed with a normal diet (ND), high-fat diet (HFD), or HFD supplemented with 200 (H200), 400 (H400), or 800 (H800) mg/kg BW HON for 8 weeks. The results showed that the mice fed HFD plus HON had lower body fat ratios (BFRs) and smaller adipocyte diameters in the epididymal WAT compared with those of the HFD group. With a proteomics analysis, the HON group upregulated 30 proteins and downregulated 98 proteins in the epididymal WAT of mice, and the steroid O-acyltransferase 1 (SOAT1) was screened as a key protein. The HON supplement prevented HFD-induced adipogenesis by reduced the mRNA and protein expression of SOAT1 and CCAAT/enhancer-binding protein-α (C/EBPα), suggesting that SOAT1 might play an important role in regulating adipogenesis. Moreover, HON treatment increased the expression of proteins related to the classical pathways of energy and lipid metabolism, such as AMP-activated kinase (AMPK) and acetyl-CoA carboxylase (ACC), and promoted the browning of epididymal WAT by upregulation of the protein expression of uncoupling protein 1 (UCP1) in the HFD mice. In conclusion, these results suggest that HON supplements could prevent increases in body fat for HFD mice by suppressing adipogenesis and promoting WAT browning.

## 1. Introduction

Obesity has become a public health issue, generally increasing in the past few decades, which is often associated with a variety of metabolic disorders such as type 2 diabetes, dyslipidemia, and cardiovascular disease [1]. The essence of obesity was believed to the growing size of adipose tissue due to excessive energy intake, secularly resulting in body fat accumulation [2]. Adipose tissue is a metabolic organ in the regulation of whole-body energy homeostasis, receiving excess energy and mobilizing stored energy [3]. In mammals, two major types of adipose tissue were previously identified: WAT and brown adipose tissue (BAT). The WAT functions as a key energy reservoir for other organs, whereas the brown adipose tissue dissipates chemical energy as heat [4]. BAT mitochondria are distinct from their counterparts in WAT in that ATP production is not their primary physiologic role. They are equipped with a specialized protein known as uncoupling protein 1 (UCP1) [5]. Reacting to certain stimuli, browning occurs in WAT, with beige or ‘brite’ (brown in white) adipocytes being recognized [6]. The UCP1 content of beige adipocytes, estimated on the basis of their abundance in the cell mixture, were similar to those of brown adipocytes, revealing that inducible beige adipocytes have a potent thermogenic ability comparable to classical brown adipocytes [7]. Therefore, inducing WAT browning could be a potential strategy to attenuate increases in body fat.

Several extractives from plants, such as capsaicin, resveratrol, curcumin, and berberine, have been reported to ameliorate metabolic disorders by improving the thermogenic ability of BAT and promoting brown remodeling of WAT [8,9,10], and research on pharmacological and nutritional agents aimed to promote WAT browning. Recently, honokiol (HON), extracted from Chinese herbal medicine, has been proven to protect against related metabolic disorders [11]. Similar to other plant extractives, such as resveratrol, HON could eliminate free radicals, decrease cancer metastasis, and inhibit bacterial proliferation [12]. In addition, HON protects skin cells against inflammation, collagenolysis, apoptosis, and senescence caused by cigarette smoke damage [13]. What is more, HON could regulate macrophage polarization by activating peroxisome, thus alleviating nonalcoholic steatohepatitis in the mice fed with a high-fat diet, which meant that HON may also affect the energy metabolism of animals [14].

Besides that, there have been many effects of honokiol on adipocyte metabolism and function reported already, particularly those suggesting a potential effect on obesity. These effects include enhanced activity inducing apoptosis [15,16] and an increase in adipocyte differentiation and insulin-stimulated glucose uptake [17], which seem to be explained by the ability of honokiol to act as a PPARγ agonist [18]. In addition to the in vitro effects of honokiol, studies in vivo have already analyzed the potential effects on adiposity and glucose homeostasis. Thus, in the same animal model used in the present work, Kim et al. [19] demonstrated that long-term honokiol supplementation mitigates body fat accumulation, insulin resistance, and adipose inflammation. As importantly, such effects were associated with increases in energy expenditure and adipose fatty acid oxidation and decreases in fatty acid synthase activity and the expression of genes related to fatty acid synthesis, desaturation, and uptake, as well as adipocyte differentiation in WAT. Moreover, the browning potential was also described in cultured adipocytes [16]. However, whether HON could prevent increases in body fat by regulating adipogenesis or the browning of WAT was not fully studied.

In this paper, differentially expressed proteins were detected before and after HON treatment by using proteomic techniques, and classical pathways related to energy metabolism and lipid metabolism were researched, aiming to study whether HON supplementation could help to resist obesity and promote WAT browning in mice with a high-fat diet.

## 2. Materials and Methods

### 2.1. Materials

Honokiol (3,3′-diallyl-4,6′-dihydroxybiphenyl (HON), purity >99.9%, Vickie Biotechnology Co.), BCA protein assay reagent (Beyotime), an RT-PCR kit (Takara, Otsu, Shiga, Japan), polyvinylidene difluoride (PVDF) membrane (Millipore, Billerica, MA, USA), and chemiluminescence (ECL) reagents (Millipore, Billerica, MA, USA) were used, and a basic diet (10% calories from fat), and a high-fat diet (60% calories from fat) were purchased from Research Diets Inc. (New Brunswick, NJ, USA).

### 2.2. Animals and Treatments

Sixty male C57BL/6J mice (6 weeks old), purchased from Hunan SJA Laboratory Animal Co., Ltd. (Hunan, China), were randomly divided into 5 groups, according to the principle of no difference in weight, with 12 mice in each group.

The mice in the different groups were provided a normal chow diet (ND group, D12450, 10% kcal from fat, 3.85 kcal/g, Research Diets Inc., New Brunswick, NJ, USA), high-fat diet (HFD group, D12492, 60% kcal from fat, 5.24 kcal/g, Research Diets, Inc., USA), or a high-fat diet plus 200 (H200 group), 400 (H400 group), or 800 mg/kg BW (H800 group) for 8 weeks. The mice were given ad libitum access to water and feed every day and kept at a constant temperature (24~26 °C) and a light/dark cycle of 12 h. After feeding for 8 weeks, the mice were weighed and executed. Adipose tissues, including shoulder blade BAT and epididymal, inguinal, perirenal, and mesenteric WAT, were washed with normal saline and weighed and then stored at −80 °C for analysis.

The ratio of all the above adipose tissues’ weight to body weight was the body fat ratio (BFR), which described the level of fat. The animal experimental protocols were conducted according to the Institutional Animal Care and Use Committee of Hunan Agriculture University (Changsha, Hunan, China).

### 2.3. Histological Analysis

The adipose tissue was rinsed with normal saline and fixed with 4% polyformaldehyde. Fixed tissues were made into 4 µm sections after being embedded in paraffin. The slices were rinsed 3 times for 5 min each time in 0.01 mol/L PBS and saline, then stained with hematoxylin and eosin (H&E) for general morphological observations. Photoshop CS6 (Adobe Systems Inc., San Jose, CA, USA) was used for quantitating the adipocyte sizes of the WAT.

### 2.4. Preparation of Protein Samples

All samples were homogenized in a lysis buffer (4% SDS, 1 mM DTT, 150 mM Tris-HCl pH 8.0, protease inhibitor). After 3 min of incubation in boiling water, the homogenate was sonicated on ice. The crude extract was then incubated in boiling water again and clarified by centrifugation at 16,000× *g* at 25 °C for 10 min. The protein content was determined with the BCA protein assay reagent (Beyotime). The supernatants were stored at −80 °C until use and prepared for proteomics analysis and Western blot analysis.

### 2.5. Proteomics Analysis

After protein digestion and TMT labeling, the peptides were fractionated by SCX chromatography using the AKTA purifier system (GE Healthcare, Little Chalfont, Buckinghamshire, UK). Each fraction was concentrated by vacuum centrifugation and reconstituted in 40 µL of 0.1% (*v/v*) trifluoroacetic acid. Then, all samples had LC-MS/MS analysis performed on them with an Orbitrap Fusion mass spectrometer that was coupled to Easy nLC (Thermo Fisher Scientific, Waltham, MA, USA). Two-dimensional polyacrylamide gel electrophoresis tests were performed. Protein spots with more than a 1.5-fold change in density (paired Student’s *t*-test yielding *p <* 0.05) and with consistent increases or decreases (upregulation more than 1.5 times or downregulation less than 0.67 times) were considered differentially expressed and were selected for further identification via MALDI-TOF-MS/MS analysis. Final bioinformatics was accomplished on a multi-omics data analysis tool named Omicsbean according to the database of the Gene Ontology (GO) program Blast2GO and KEGG (Kyoto Encyclopedia of Genes and Genomes). Details regarding the immobilized pH gradient (IPG)-2-DE and image analysis, as well as the MALDI-TOF-MS/MS analysis, can be found in the Supplementary Materials. All experiments were performed at least in triplicate to ensure reproducibility.

### 2.6. RT-qPCR Analysis

The total RNA was extracted from the epididymal WAT using an RNA extraction kit (Accurate Biotechnology, Hunan, China). The cDNA was synthesized by a TIANScript RT kit (Tiangen Biotech, Beijing, China). In strict accordance with the instructions of the SYBR kit (Takara, Otsu, Shiga, Japan), RT-qPCR was performed on the Techne Quantica RT-PCR detection platform (Techne, Staffordshire, UK). The PCR sequence of each primer is shown in Table 1.

### 2.7. Western Blot Analysis

The protein was extracted from the epididymal adipose tissue and quantified by an enhanced BCA protein assay kit (Beyotime Biotech Inc., Shanghai, China). Then, SDS gel electrophoresis was carried out, and the bands were visualized with extreme hypersensitivity ECL chemiluminescence kit reagents (Beyotime Biotech Inc., Shanghai, China). The relative amount of proteins associated with specific antibodies was quantified with Lumi Vision imager software.

### 2.8. Statistical Analysis

All data were presented as means ± SEM. SPSS 21.0 (SPSS Inc., Chicago, IL, USA) was used for one-way ANOVA, followed by Duncan corrections, and *p <* 0.05 was considered statistically significant.

## 3. Results

### 3.1. HON Mitigates the Body Fat Ratio and Average Adipocyte Diameter in HFD-Induced Mice

**Figure 1:**
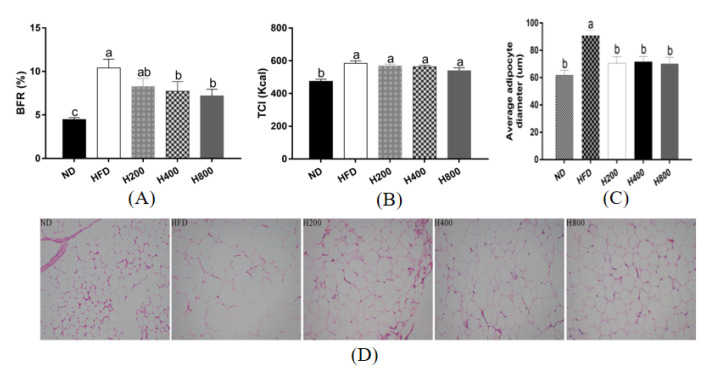
Effect of HON on the BFR and average adipocyte diameter in the epididymal of HFD-fed mice. The mice received either a normal diet (ND), high-fat diet (HFD), or high-fat diet with HON at 200 (H200), 400 (H400), or 800 mg/kg BW (H800) for 8 weeks. Changes in the (**A**) body fat ratio (BFR) and (**B**) total calorie intake (TCI) during the trial period (BFR = total adipose tissues’ weight (g)/body weight (g); TCI = total food intake during trial period (g) × unit energy (kcal/g)). (**C**) Average adipocyte diameter of epididymal WAT at × 200 magnification. (**D**) The epididymal WAT was dissected for morphological observations at 200×. Means ± SEM (*n* = 6). The different letters above the columns were statistically significant (*p* < 0.05).

As shown in Figure 1, compared with the ND group, the final average BFR of the HFD group significantly increased *(p <* 0.01), indicating that the high-fat diet had induced obesity. Compared with the HFD group, the BFR in H200 was insignificantly reduced by 20.56% *(p >* 0.05), whereas it was significantly reduced by 25.74% and 30.93% in the H400 and H800 groups *(p <* 0.05), respectively (Figure 1A). The caloric intake of each group was also evaluated. Compared with the HFD group, HON (200, 400, and 800 mg/kg) treatment did not significantly decrease the total calorie intake (TCI), suggesting that the lowering of the BFR was not caused by the decrease in energy intake (Figure 1B).

To further study whether the decreased BFR was related to the reduction in adipose tissue mass, the epididymal WAT was dissected for morphological observations. The HFD group had significantly increased adipocyte diameters, but this result was reversed by adding 400 or 800mg/kg HON (Figure 1C). The fat mass was associated with the BFR in the HON supplement group, suggesting that the BFR being decreased by the HON supplement could be attributed to adipocyte diameter reduction. Compared with the HFD group, HON treatment decreased the epididymal WAT adipocyte diameter significantly (Figure 1D), indicating that HON may inhibit the hyperplasia of WAT.

### 3.2. HON Influences the Expression of Proteins in the Adipose Tissue of HFD-Fed Mice

**Figure 2:**
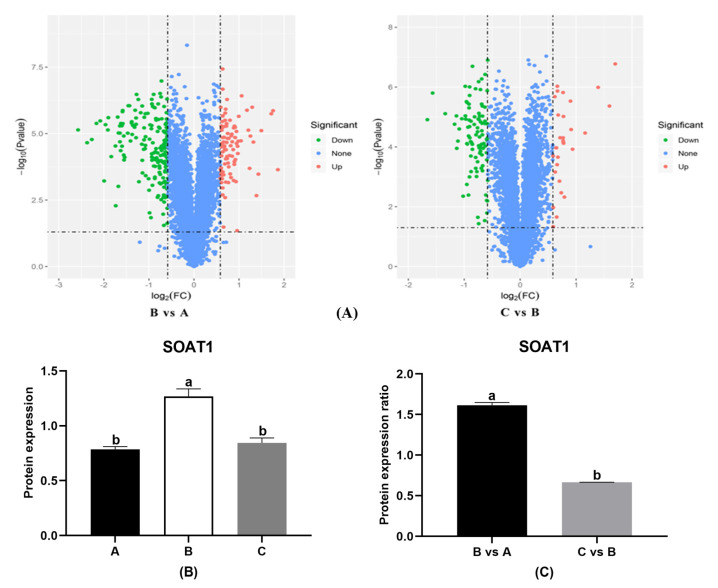
Proteomic results of HON treatment in HFD-fed mice. (**A**) The protein volcanic map of HON treatment in HFD-fed mice. Protein spots were screened by the criterium of a more than 1.5-fold change in density (paired Student’s *t*-test yielding *p <* 0.05) with consistent increases or decreases. The volcano plot was drawn on the basis of the fold change (FC) of proteinic expression and the P-value obtained from the T test to describe the difference between two groups. “A” = ND group; “B” = HFD group; and “C” = HON treatment. The transverse coordinate is the FC (logarithmic transformation based on 2), and the longitudinal coordinate is the *p*-value (logarithmic transformation based on 10). Red spots reflect the upregulated proteins (upregulation more than 1.5 times and *p* < 0.05), whereas green spots reflect the downregulated proteins (downregulation less than 0.67 times and *p* < 0.05) and blue spots constitute the proteins with no significant difference. (**B**) Expression of SOAT1 (differentially expressed proteins) in each group. (**C**) The expression ratio of SOAT1 for B vs. A and C vs. B. The different letters above the columns were statistically significant (*p* < 0.05).

In order to screen for different proteins which might be associated with the browning of adipose tissue, we used proteomics analysis. Based on the data of the BFR, samples from H800 were chosen to represent HON treatment. Samples from the ND group, HFD group, and HON treatment were labeled “A”, “B”, and “C”, respectively. There was a total of 5130 proteins identified in the adipose tissue of the epididymis (Table 2). The fold change (FC) of proteinic expression and the *P*-value obtained from the T test were used to draw a volcano plot (B/A, C/B) describing the difference between the two groups (Figure 2A). According to Figure 2A, the HFD group significantly upregulated 94 proteins and downregulated 183 proteins compared with the ND group. Compared with the HFD group, the numbers of upregulated proteins and downregulated proteins in the H800 group were 30 and 98, respectively. Steroid O-acyltransferase 1 (SOAT1), a protein catalyzing the conversion of free fatty acids and cholesterol into cholesterol esters, was found to have significantly different expression among the three groups (*p* < 0.05) (Figure 2B, Table S1) and different expression ratios between B vs. A and C vs. B (Figure 2C).

### 3.3. HON Affects the Epididymal WAT Browning-Related Gene Expression in HFD-Fed Mice

**Figure 3:**
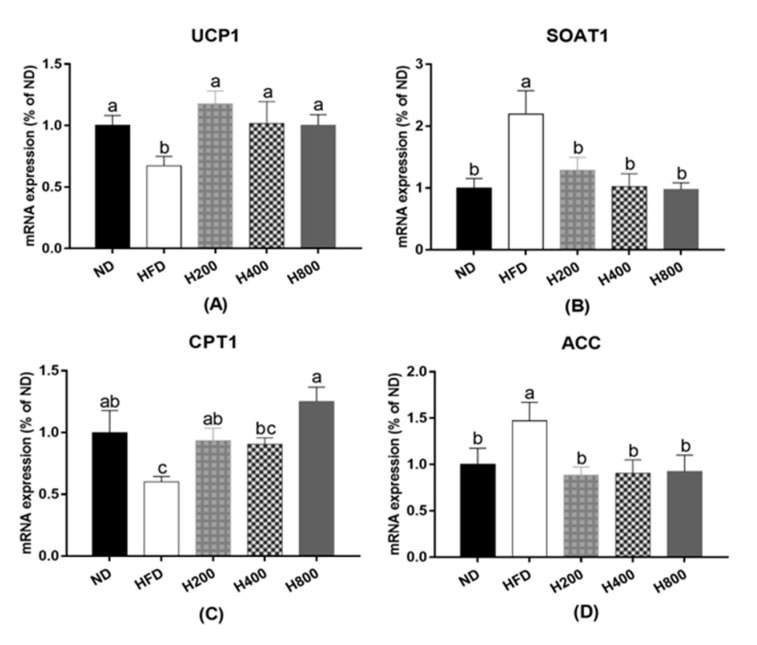
Effects of HON on brown remodeling-related mRNA expressions in epididymal WAT. RT-PCR was used to detect the relative mRNA expression of adipogenic genes relative mRNA expression of UCP1 (**A**), SOAT1 (**B**), CPT1 (**C**), ACC (**D**). The different letters above the columns were statistically significant (*p* < 0.05).

To investigate whether the expression of browning related genes in HON-regulated epididymal WAT browning was detected by RT-PCR analysis, we compared the ND group with the mRNA expression of upregulated SOAT1 and significant downregulation of CPT1A, ACC, and UCP1 in the HFD group (*p <* 0.05), as shown in Figure 3. HON treatment significantly inhibited mRNA expression of SOAT1 and significantly increased mRNA expression of CPT1A, ACC, and UCP1 (*p* < 0.05).

### 3.4. HON Regulates the Epididymal WAT Browning-Related Protein Expression in HFD-Fed Mice

**Figure 4:**
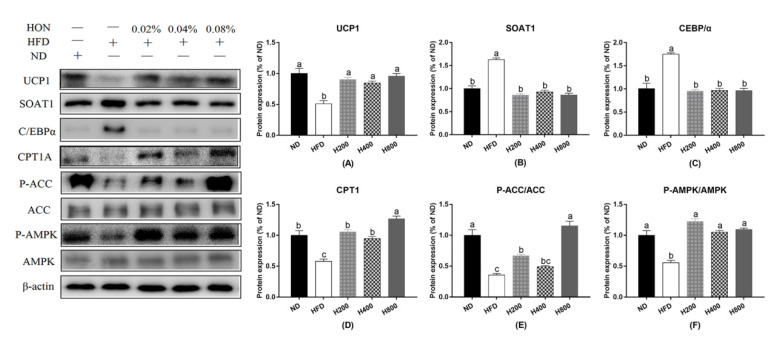
Effects of HON on brown remodeling-related protein expressions in epididymal WAT. Relative protein levels of UCP1 (**A**), SOAT1 (**B**), CEBP/α (**C**), CPT1A (**D**), P-ACC/ACC (**E**), P-AMPK/AMPK (**F**) were measured by Western blot analysis. The different letters above the columns were statistically significant (*p* < 0.05).

As for the results of the Western blot analysis, the relative protein levels of SOAT1 and C/EBPα were significantly upregulated in the high-fat diet (*p <* 0.05), whereas the relative protein levels of P-ACC/ACC, P-AMPK/AMPK, CPT1A, and UCP1 were significantly decreased (*p <* 0.05). HON treatment significantly inhibited the protein expression of SOAT1 and C/EBPα (*p <* 0.05) in the epididymal WAT of the mice that were fed a high-fat diet and promoted the protein expression of P-ACC/ACC, P-AMPK/AMPK, CPT1A, and UCP1 significantly (*p <* 0.05) (Figure 4).

### 3.5. HON Increases the Expression of UCP1 in the Inguinal WAT of HFD-Fed Mice

**Figure 5:**
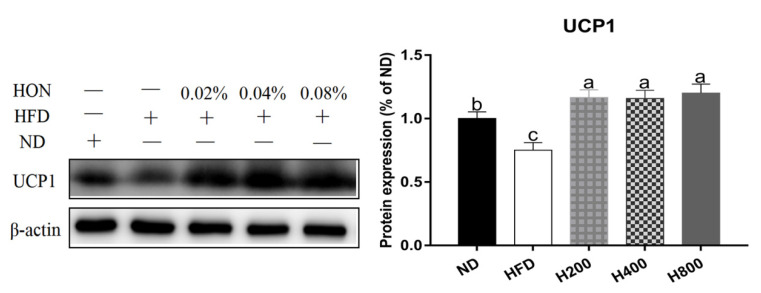
Effects of HON treatment on UCP expression in the inguinal WAT. Relative protein levels were measured by Western blot analysis. The different letters above the columns were statistically significant (*p* < 0.05).

To further confirm whether HON promoted brown remodeling, the contents of UCP1 were assessed in the inguinal WAT. As shown in Figure 5, the expression of UCP1 in the inguinal WAT in the HFD mice was significant compared with the ND group (*p* < 0.05), whereas HON treatment restored the expression of UCP1.

## 4. Discussion

Obesity has become a public health issue, as we know, and the browning of white adipose tissue can change the body’s energy distribution and improve the disorder of energy metabolism so as to achieve beneficial effects on the body. Many studies have researched the pharmacological and nutritional agents aimed at promoting WAT browning, which are of interest for combatting obesity. To date, the induction of WAT browning by dietary agents or nutrient derivatives has been reported for a wide variety of food bioactive ingredients, including polyphenols. The ability of nutrient derivatives to induce UCP1 expression in WAT was reported earlier by the administration of n-3 PUFA [20], fucoxanthin [21], retinoids [22], 2-hydroxyoleic acid [23], and bofutsushosan [24], among others. Honokiol might share some of the molecular mechanism for inducing UCP1 expression in white adipocytes described by several of the above-mentioned compounds. Interestingly, honokiol acts as a PPARγ agonist [18] and as a rexinoid X receptor ligand [25]. In addition, excessive energy intake was synthesized to FFA in the liver, which entered the WAT to be stored as a form of fat, resulting in expansion of the adipose tissue. Specific stimuli, such as cold stress, reactive oxygen species (ROS), and plant extracts (such as catechin or resveratrol), could induce WAT to undergo browning [26,27,28]. The process of browning depends on the expression of UCP1 in the mitochondria of WAT [29]. UCP1 eliminated the proton concentration gradient across the membrane on both sides of the mitochondrial inner membrane and removed the chemical coupling between the electron transfer chain and oxidative phosphorylation in the respiratory chain, by which the production of adenosine triphosphate (ATP) was inhibited [30]. The expression of UCP1 in brown WAT could resist obesity and metabolic dysfunction by dissipating chemical energy as heat [31]. In the present study, the HON supplement (400 mg/kg and 800 mg/kg) mitigated the BFR gain without influencing the food intake of mice fed with an HFD, restrained the hyperplasia of the epididymal WAT, and upregulated the expression of UCP1 in the epididymal and inguinal WAT. These results suggest that HON supplementation could inhibit adipogenesis, promote WAT browning, and then reduce obesity.

With more than a 1.5-fold change in density from the proteomics analysis, SOAT1, a key enzyme that catalyzes free cholesterol and free fatty acids to form cholesterol esters in endoplasmic reticulum and then stores them in lipid droplets, was screened as a key protein. In the results of the RT-PCR and Western blot analyses, the HFD increased the mRNA and protein expression of SOAT1 and protein expression of CCAAT/enhancer-binding protein-α (C/EBPα) in the epididymal adipose tissue of mice, which could be reversed by HON treatment. C/EBPα is an important transcription factor for promoting adipogenesis in the early stages of adipocyte differentiation and promoting adipogenic differentiation and fat accumulation [32,33]. The C/EBPα-derived promoter sequence is present on SOAT1, and insulin treatment increases the binding of C/EBPα on the sequence of the SOAT1 promoter [33]. A high-fat diet could overexpress C/EBPα and cause low expression in the WAT of C/EBPα–deficient mice [33,34]. These findings suggest that HON supplementation could inhibit adipogenesis by suppressing C/EBPα and SOAT1 expression.

AMP-activated kinase (AMPK), which plays an important role in regulating cell energy homeostasis and lipid metabolism [35], is a major energy sensor that integrates signals from nutrition, hormones, and stress to maintain cellular energy homeostasis [36]. The activation of AMPK could improve the imbalance of energy metabolism and maintain the homeostasis of energy metabolism by enhancing the browning of white adipocytes [37]. In a recent report, a milk fat globule membrane (MFGM) supplement significantly reduced obesity by suppressing the PPARγ and C/EBPα proteins and mRNA expression and activating the AMPK pathway [38]. Acetyl-CoA carboxylase (ACC) catalyzes the production of malonyl-CoA, which is a rate-limiting enzyme for the synthesis of fatty acids [39]. AMPK phosphorylation could suppress lipid synthesis by deactivating ACC in the adipose tissue, which was named the AMPK/ACC pathway [40,41]. HON treatment promoted protein expression of P-AMPK/AMPK and P-ACC/ACC, showing its activation on the AMPK/ACC pathway and the inhibitory effects on adipogenesis.

The ability of HON to act as an RXR ligand suggests that it could enhance the transcriptional activity of genes that present a retinoic acid responsive element (RARE) within their promoter sequences. Among other receptors, RXR can bind to a retinoic acid receptor (RAR), and this heterodimer controls the expression of many genes through RARE, including the UCP1 gene. As was previously suggested, the anti-obesity effects of honokiol could rely on the reduction in fatty acid synthesis in the WAT. Similarly, retinoic acid decreases C/EBPα expression and the triglyceride content, in addition to inducing CPT1 expression and fatty acid oxidation in mature white adipocytes [21] and UCP1 protein expression in other white adipocyte models [42]. Moreover, glucose homeostasis is particularly relevant, given HON’s ability to act as a PPARγ ligand. HON directly triggered the increase in PPARγ activity, and the mobilization of fatty acid esters indirectly caused the increase in PPARγ activity, which led to the induction of UCP1 in white adipocytes. However, whether HON could prevent increases in body fat by regulating glucose and lipid homeostasis is the limitation of this study.

**Figure 6:**
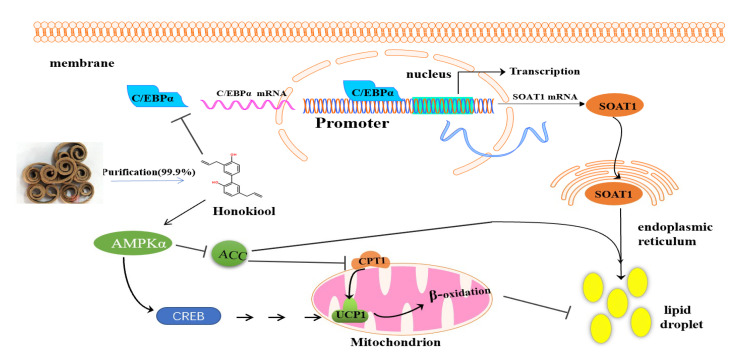
Schematic diagram of the underlying molecular mechanism of the WAT browning effect of HON in high-fat-induced body fat gain. HON treatment effectively inhibits the high-fat diet-induced C/EBPα and SOAT1 and activates P-AMPK, P-ACC, CPT1, and UCP1 expression (black arrow), which could inhibit adipogenesis.

HON also enhanced the expression of UCP1 in the epididymal and inguinal WAT of the HFD mice. Activation of the AMPK pathway could decrease the production of malonyl-CoA, which is an inhibitor of CPT1 (a rate-limiting enzyme that transports fatty acids between mitochondrial membranes for β-oxidation) [43]. Moreover, the fatty acids and ROS produced from β-oxidation could induce the expression of UCP1 [44,45], showing that CPT1 may upregulate UCP1 expression. In the present study, the expression of CPT1 and UCP1 was positively correlated, which was consistent with the experimental results of Lone and Yun [16]; that is, HON supplementation could increase the protein expression of CPT1 and UCP1 in 3T3-L1 cells, suggesting that HON may induce the AMPK-ACC-CPT1 pathway to achieve the browning of WAT in HFD mice. In addition, HON supplementation could inhibit adipogenesis by suppressing C/EBPα and SOAT1 expression and activating the AMPK-ACC-CPT1 pathway, resulting in the relative content of fatty acid entering the adipose synthesis pathway decreasing while the relative content entering β-oxidation relatively increases in the WAT, which is consistent with the Western blot and RT-PCR analysis results and which might affect the expression of UCP1. The findings obtained in the present study are summarized briefly in Figure 6. However, the exact mechanism remains to be studied further.

## 5. Conclusions

The HON supplement reduced obesity in the HFD mice by suppressing adipogenesis and promoting WAT browning. In this process, the downregulation of SOAT1 by HON might play an important role in adipogenesis, while the upregulation of AMPK-ACC-CPT1 by HON might also take the synergistic reaction into account, but the specific mechanism needs to be studied further.

## Figures and Tables

**Table 1:** Primer sequences used for real-time quantitative PCR.

Genes	Primer Sequence (5′–3′)	
UCP1	Forward	ACTGCCACACCTCCAGTCATT
	Reverse	CTTTGCCTCACTCAGGATTGG
SOAT1	Forward	AACTCCATCTTGCCAGGTGTCTTG
	Reverse	ACCACGTTCCAGGTCCTGTAGTAG
ACC	Forward	TTGAAGGCACAGTGAAGGCTTACG
	Reverse	GACGCCATCTTCCTCTGTCAGTTG
CPT1A	Forward	GTGGCATCTCCTTTAACTCAAC
	Reverse	CGGCGTTGAAGATCTTGTATTC
β-actin	Forward	GACATTTGAGAAGGGCCACAT
	Reverse	CAAAGAGGTCCAAAACAATCG

**Table 2:** Statistics of the protein identification results.

Database ^1^	Tatal Spectra ^2^	Spectra (PSM) ^3^	Peptides ^4^	Protein Groups ^5^
Mus musculus	364220	68436	30981	5130

Database ^1^: the database species name to use. Tatal spectra ^2^: total number of secondary mass spectrograms. Spectra (PSM) ^3^: peptide spectrum match, identifying the number of peptides to match. Peptides ^4^: total number of identified peptides. Protein groups ^5^: total number of identified proteins.

## Data Availability

The study did not report any data.

## Supplementary Materials

The following are available online at https://www.mdpi.com/article/10.3390/ani11061493/s1, Table S1: Difference genes and accession statistics.
