# Supplementary material for: Honokiol Alleviates High-Fat Diet-Induced Obesity of Mice by Inhibiting Adipogenesis and Promoting White Adipose Tissue Browning
Source: Animals (Basel). 2021 May 21;11(6):1493. doi: 10.3390/ani11061493 (PMC8224378; doi:10.3390/ani11061493)
Supplement: Supplementary file 1 [file animals-11-01493-s001.zip › animals-1208459-supplementary.pdf]

Supplementary Table

Table S1. Difference genes and accession statistics.

Accession	Gene names	Accession	Gene names	Accession	Gene names
A7E2R1	Amh	Q545B6	Stmn1 mCG_12955	E9Q634	Myo1e Myr3
P56564	Slc1a3 Eaat1 Gmt1	Q9WUU7	Ctsz	E9PV44	ATPif1
Q7TPZ8	Cpa1	P29452	Casp1 Il1bc	Q91XV3	Basp1 Nap22
P40936	Inmt Temt	P08030	Aprt	Q3TC82	Ncf4
Q64523	Hist2h2ac Hist2h2ab	Q3UBR4	Ctss	Q69ZV3	mKIAA0930
A0A1B0GS05	Gm6657 mCG_52111	P18242	Ctsd	Q8VBT6	Apobr Apob48r
B2RS76	Cpb1 mCG_21890	Q91VW3	Sh3bgrl3	S4R293	Ncf1
D3Z6P0	Pdia2 Pdip	A0A0G2JGD2	S100a4	Q6PB99	Evl
Q3UDM8	Rpe	P56654	Cyp2c37	Q9CQI6	Cotl1 Clp
B2RUJ4	Atad5	G3UXL2	Prps1l3	Q3V471	Lgals3
Q91X79	Cela1 Ela1	E9PUA7	Tpd52	Q52KE3	Gnl1 Gna-rs1 Gnal1
A0A2I3BPM4	Sdr39u1	Q9JHF7	Hpgds Gsts Pgds PTGds2	Q3UN10	Wfs1
Q3U4N8	Csf1r	Q542I8	ITGb2 mCG_3383	Q3U6N7	Lcp1
O08745	Adgre1 Emr1	D3YZ86	Rab3il1	Q8BYW1	Arhgap25 Kiaa0053
Q80SY3	ATP6v0d2	Q3U3E5	Msr1	Q9ESY9	Ifi30 Gilt Ip30
O89017	Lgmn Prsc1	A0A1W2P8C6	Cd63	Q52L78	Cryab mCG_4166
A0A087WNN8	SOAT1	F7CVJ5	Ahnak2	Q3UJ44	Capg
Q3UND0	Skap2 Prap Ra70 Saps Scap2 Skap55r	E9M0G2	Lep	A0A0G2JGC2	Adgrl2
Q6TCG2	Paqr9	Q04447	Ckb Ckbb	Q542W1	Il1rn mCG_4837
A0A0G2JDJ6	Snx8	P45377	Akr1b8 Fgfrp	Q3TVP0	mCG_12056 3
Q7M6Y6	Mroh2b HEatr7b2	P31996	Cd68	Q2TBE6	Pi4k2a
A0A0N4SV76	Chmp3	F6QWW7	Wipf1	Q3UE75	Gpnmb
P12265	Gusb Gus Gus-s	O35405	Pld3 Sam9	Q3TJ69	Serpinb9b
Q9DB10	Smdt1 Emre	Q14AX9	Mrc2 mCG_4815	Q3TCW6	Mmp12
Q62426	Cstb Cst6 Stfb	Q8BLF1	Nceh1 Aadacl1 Kiaa1363		
